# Comparative Approach of Tracking COVID-19 in Balkan Countries Using Interactive Web-Based Dashboard

**DOI:** 10.1155/2022/8627956

**Published:** 2022-12-21

**Authors:** Oriana Zaçaj, Etleva Beliu, Endri Raço, Kleida Haxhi, Kostaq Hila

**Affiliations:** Polytechnic University of Trane, Mother Teresa Sq.4, Tirana, Albania

## Abstract

**Objective:**

The design and implementation of an online dashboard to support data-driven decision-making and joint coordination between health institutions and government bodies in the Balkan countries faced with new COVID-19 waves in the region.

**Methods:**

Shiny R dashboard tracks COVID-19 in real-time using a comparative approach to interactively visualize national-level data from various official sources.

**Results:**

The dashboard, named COVID-19 Situation in Balkan countries can be accessed online (COVID-19 Situation in Balkan countries). The daily situation in 11 Balkan countries focuses on similarities and differences between countries on a daily basis and since the beginning of the pandemic. The web resource features the most affected countries, the number of new cases, and fatality rates reported daily. Features also include rankings of the worst affected countries, information search and filtering, and a map component interactively showing daily information for each country comparatively.

**Conclusions:**

The dashboard for the COVID-19 situation in Balkan countries simplifies meaningful real-time information for public and health agencies regarding the COVID-19 situation in the whole Balkan region. The creation allows for a deep analysis of measures taken to face COVID-19 in a regional context, allowing for health policy updates and a better basis for collaboration among Balkan governments.

## 1. Introduction

Since its start in December 2019, COVID-19 has killed more than six million people worldwide, and the number of fatalities is increasing every day [1]. Balkan countries faced COVID-19 in early 2020, and measures initially taken by the government seemed to have a positive effect on preventing the pandemic's spread. Nevertheless, the region has unfortunately been severely affected by the COVID-19 waves that are still going on. Although the pandemic appears to be gradually returning to endemic status, official data show a disturbing reality. North Macedonia, Bosnia and Herzegovina, and Serbia have recently led the list of European countries with the highest infection rate. Bosnia has seen an increase of 78% in new cases, followed by Serbia with 75%, according to the Worldometer [2].

The governments of Balkan countries are clearly failing to control the spread of COVID-19, mainly due to their visible hesitation in reinstating safety measures. Other factors, such as the seasonal mobility of people within the region and the resistance of individuals to vaccinations, contribute to an increasing number of hospitalizations for patients who demonstrate complications and require intensive care [3–5].

In this confusing situation, when COVID-19 is still a dangerous enemy but social isolation is hopefully a thing of the dark past, vaccination is still a choice, and mobility restrictions are a great burden for the fragile Balkan economies, it is crucial to call for regional cooperation. Coordinated decisions between Balkan governments seem to be the most important key to battling the pandemic's spread. As decisions should be fast and data-driven, technical health experts have to be in real-time informed about disease key indicators in the population and how these indicators compared to those in neighbouring countries in the region. Another cornerstone of this cooperation is that the information collected should be shared as quickly as possible with all offices and agencies responsible for public health in the Balkans.

For all these reasons and more, the aim of this paper is to provide a web-based tool to track real-time COVID-19 in the Balkan region and display the retrieved data in an interactive and easy-to-interpret way.

While there are many web resources providing information regarding COVID-19 at a national-data level [6, 7], we feel there is a gap regarding existing dashboards with insights regarding the pandemic situation and comparative views in the context of the Balkan region as a whole.

The objective of this study is to offer a simple-to-use yet powerful tool for making information more accessible and interpretable to health agencies in charge of controlling the pandemic spread and supporting data-driven decision-making and joint coordination between health institutions and government bodies in the Balkan countries faced with new COVID-19 waves in the region.

Apart from offering this tool to governments and health institutions as an innovative way of monitoring and taking data-driven decisions regarding the COVID-19 situation in Balkan countries, this research aims to create a larger academic discussion on the benefits of open-source tools applied in medicine and the usage of such tools in teaching and educating future generations of health professionals.

## 2. Methods

### 2.1. Implementation

To make the comparison of COVID-19 situations in Balkan countries possible, we have created an online system called COVID-19 Situation in Balkan countries, accessible from https://endriraco.shinyapps.io/BalkanCovidSituation/?_ga=2.209755413.1493368631.1662490142-405543965.1659781086. The work for this research started immediately after the first cases of COVID-19 were registered in Balkan countries in March 2020 and ended with the creation of the dashboard in June 2022.

### 2.2. Software Used

The web source was created by combining free and open-source tools. R development environment for statistical computing and graphics, the Shiny platform based on R for building websites, and various R libraries, as listed in Table 1.

The R language is a free and open-source statistical software that offers tremendous potential when it comes to data wrangling, analysis, visualization, and reporting [8]. The editor of choice to facilitate the coding process was RStudio Desktop, Open-Source Edition [9], due to its excellent set of integrated tools designed to increase productivity with R. The web framework for building our dashboard was Shiny [10], an open-source R package that enabled the user to start developing dashboards without much effort, focusing on the user experience rather than the designing process. The starting prototype for this dashboard was set up using the ShinydashboardPlus package [11], an extension of the package Shinydashboard [12], which permits users to enrich their dashboard with additional functions. National-level data for the dashboard were made possible from different official sources, using the Covidregionaldata R package as an interface [13]. Through this interface, data were extracted from sources, cleaned, and saved as daily time series in data frames. Further analysis of data, their preparation for visualization, and the production of indicators were performed using the tidy verse collection of R packages, which include many various functions and excellent workflow coverage for data analysis [14]. The interactivity of graphs was provided by the plotly package [15], whilst dashboard map features were created using the leaflet package [16], supported by scales [17], RColorBrewer [18], and viridisLite [19] for improved visualization. A table view of the data was provided on the homepage using the library DT possibilities [20] so that the user could easily find the required indicator value for the date of interest.

The dashboard is organized as an R studio project based on an app. A map is an R file that contains code for the interface and functionality. R file that contains code for the map feature of the dashboard and data. R file that contains code for data download and preparation. The entire project and files are uploaded to GitHub and can be freely accessed using the link https://github.com/endri81/BalkanCovidDashboard.

### 2.3. Interface and Functionality

Dashboard COVID-19 Situation in Balkan countries (https://endriraco.shinyapps.io/BalkanCovidSituation/?_ga=2.209755413.1493368631.1662490142-405543965.1659781086) is publicly available, can be accessed in any browser, and is updated automatically on daily basis. The single-screen user interface is organized into three logical sections.

#### 2.3.1.  Section 1: Significant Metrics

This section contains widgets containing information about the countries registering the highest values of COVID-19 metrics, as follows: daily new cases; daily fatalities; and the number of fatalities since the pandemic outbreak.

#### 2.3.2.  Section 2: Comparative Analysis and Spatial Representation

This section contains different graphs to compare the COVID-19 situation over time in all Balkan countries. Four-time series charts give a complete picture of differences and similarities in Balkan countries when it comes to the spread characteristics for the current year and also the progression of COVID-19 since the start of the pandemic. Daily new cases and fatalities are organized into separate plots to allow experts a more detailed analysis. Different bar charts based on user choice input allow a daily comparison of the situation in all Balkan countries. This section also contains a spatial map that represents key COVID-19 metrics for each country in an interactive comparative way.

#### 2.3.3.  Section 3: Data Filtering

This section contains updated daily data represented as a searchable, filter-capable table, allowing users to easily find information regarding the country of interest or time period. The data are obtained from different official sources such as Public Health England in the UK, the World Health Organisation (WHO), the European Centre for Disease Prevention and Control (ECDC), Johns Hopkins University (JHU), Google Open Data, etc. using Covidregionaldata as an interface to national-level COVID-19 data.

## 3. Results

Dashboard COVID-19 Situation in Balkan countries (https://endriraco.shinyapps.io/BalkanCovidSituation/?_ga=2.209755413.1493368631.1662490142-405543965.1659781086), presented in this paper, is able to highlight the COVID-19 context in the Balkan region by visualizing the pandemic spread in Balkan countries using real-time processing of data.

This web source provides information regarding the following:

- New cases and fatalities for each Balkan country from the outbreak of COVID-19 are up-to-date.
- New cases and fatalities per million people for each Balkan country from the outbreak of COVID-19 are up-to-date.
- Information on countries registering the highest values of the COVID-19 metrics.
- Real-time comparison of the COVID-19 situation based on daily new cases and fatalities.

As mentioned above, this information is presented using time series plots, maps, info boxes, bar charts, and a searchable table. The dashboard offers the functionality of directly downloading every plot or table data for the user.

### 3.1. Visualizations

**Figure 1:**
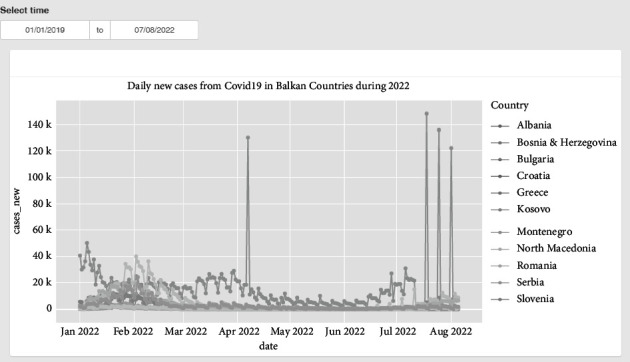
Daily COVID-19 new cases in Balkan countries during 2022.

Figure 1 illustrates the comparison of 11 Balkan countries with respect to the daily new cases of COVID-19 during the year 2022. The figure also illustrates the time selection feature offered in the dashboard, which allows for the selection of a time range of interest for the comparison.

**Figure 2:**
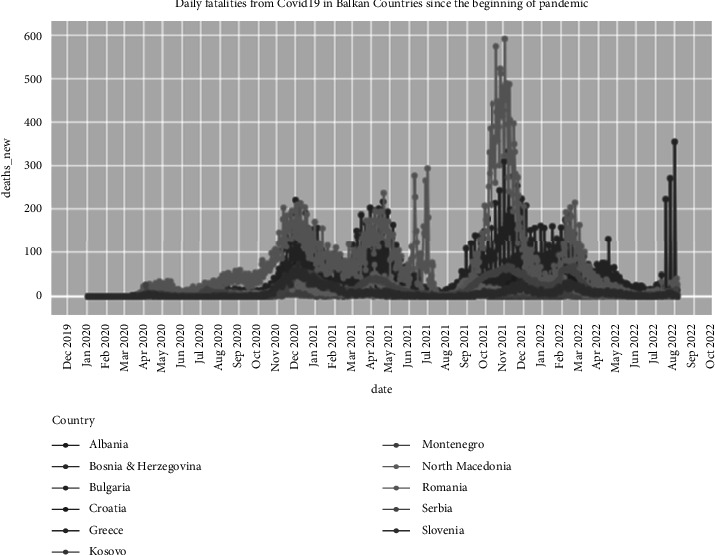
Daily COVID-19 fatalities in Balkan countries since the pandemic outbreak.

Figure 2 illustrates the comparison of eleven Balkan countries regarding COVID-19 daily fatalities since COVID-19 is up-to-date.

**Figure 3:**
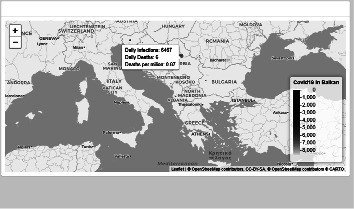
Dashboard map feature offering information regarding COVID-19 metrics.

Figure 3 illustrates the comparison of eleven Balkan countries regarding COVID-19 daily metrics represented using dashboard map functionality. On mouse over the selected country map, a label containing daily COVID-19 metrics for this country is displayed. Each country on the map has a bubble, whose color and size characterize the severity of the COVID-19 situation for this country.

**Figure 4:**
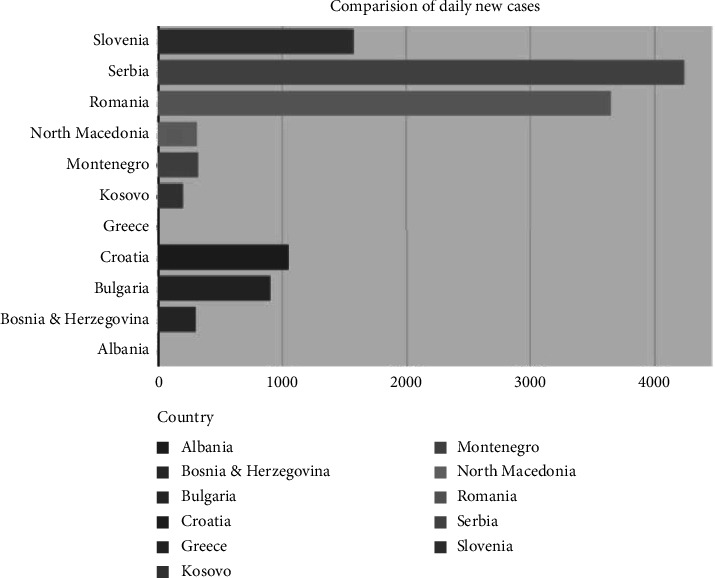
User-choice possibility to generate a bar chart on COVID-19 metrics of interest.

Figure 4 illustrates the possibilities the dashboard offers the end-user in choosing a metric of comparison for assessing the COVID-19 real-time situation in all Balkan countries.

## 4. Discussion

The results show that the dashboard presented in this document allows for comparisons of the main pandemic indicators of affected countries and is very effective. The study shows how visualization technology and health data can be combined to track COVID-19 conditions not only at the national level but throughout the region. The dashboard created provides a demonstration of the use of open-source tools to make medical indicators more intuitive and readable to users. The following are some of the conclusions drawn from the monitoring systems established in this investigation. First, the number of deaths caused by COVID-19 infection in some Balkan countries such as Romania reached its maximum in late 2021, much after the vaccine was administered by the governments of these countries [21]. Second, while Balkan countries follow similar patterns during COVID-19 waves, there are countries with large differences in COVID-19 indicator values, requiring a deeper analysis of public health institutions.

Of course, the COVID-19 models and related knowledge are developing very quickly based on the latest data. Balkan countries are limited in resources to sustain this development, meaning joint action is needed from health institutions, academics, and governments, supported by information analysis tools such as this dashboard. We are aware of the existence of many other tools to monitor the spread of COVID-19, but we also acknowledge that the tool introduced in this document is innovative in the way it offers a comparative view of the situation with COVID-19 and its region-related specifics. We also acknowledge that the application is still in continuous development to offer more possibilities, including geospatial analysis, making it possible for researchers to take environmental risk factors into consideration [22]. Our system uses data sourced from many COVID-19 data collections [13] as a real-life illustration of the possibility of making data accessible to the community. However, we realize that dashboards, as tools for tracking and monitoring epidemic situations, can suffer from data latency [23]. Our dashboard is not an exception from this problem considering moments like late reporting at the beginning of a virus outbreak, wrong data due to lack of testing, and latency of reaction from WHO [24].

## 5. Conclusions

Using open-source tools to create dashboards to track and monitor COVID-19 indicators at the country or regional level represents an easy and low-budget approach, which can be easily implemented by health institutions in countries similar to the Balkans. Such dashboards are not limited to COVID-19 but can be easily adapted to perform similarly in tracking epidemic situations for various viral diseases. This research calls for cooperation between the governments of Balkan countries in sharing the information collected as quickly as possible with all offices and agencies responsible for public health in the Balkans. The results of the research will hopefully serve as an example of how this type of cooperation can be implemented in practice by using small amounts of funds aimed at combating the epidemic to create similar tools.

## Figures and Tables

**Table 1:** Software used.

Software/library	Purpose	Version
*R*	Programming language	Version 4.2.1
Shiny	Web application framework for R	Version 1.7.2
Shiny dashboard	Create dashboards with Shiny	Version 0.7.2
ShinydashboardPlus	Add more components to Shinydashboard	Version 2.0.3
Covidregionaldata	Subnational data for Covid-19	Version 0.9.3
Leaflet	Create interactive web maps	Version 2.1.1
Tidy verse	Working with data	Version 1.3.2
Scales	Scale functions for visualization	Version 1.2.0
DT	Wrapper of javascript library data tables	Version 0.23
Plotly	Create interactive web graphics	Version 4.10.0
RColorBrewer	ColorBrewer palettes	Version 1.1–3
viridisLite	Colorblind friendly color maps	Version 0.4.0
RStudio	R environment editor	Version 1.0.143

## Data Availability

Interface at the subnational and national level COVID-19 data are sourced from both official sources, such as Public Health England in the UK, and from other COVID-19 data collections, including those of the World Health Organisation (WHO), the European Centre for Disease Prevention and Control (ECDC), Johns Hopkins University (JHU), Google Open Data, and others.
